# Surface Wiping Test to Study Biocide -Cinnamaldehyde Combination to Improve Efficiency in Surface Disinfection

**DOI:** 10.3390/ijms21217852

**Published:** 2020-10-23

**Authors:** Joana F. Malheiro, Catarina Oliveira, Fernando Cagide, Fernanda Borges, Manuel Simões, Jean-Yves Maillard

**Affiliations:** 1 LEPABE—Laboratory for Process Engineering, Environment, Biotechnology and Energy, Faculty of Engineering, University of Porto, Rua Dr. Roberto Frias, 4200-465 Porto, Portugal; up201409754@fe.up.pt; 2 Cardiff School of Pharmacy and Pharmaceutical Sciences, Cardiff University, Cardiff, Wales CF10 3NB, UK; 3 CIQUP, Department of Chemistry and Biochemistry, Faculty of Sciences University of Porto, Rua do Campo Alegre, 4169-007 Porto, Portugal; catarinaoliveirafc@gmail.com (C.O.); fernando.fagin@fc.up.pt (F.C.); fborges@fc.up.pt (F.B.)

**Keywords:** bacteria, cinnamaldehyde, cetyltrimethylammonium bromide, formulation, surface disinfection

## Abstract

Disinfection is crucial to control and prevent microbial pathogens on surfaces. Nonetheless, disinfectants misuse in routine disinfection has increased the concern on their impact on bacterial resistance and cross-resistance. This work aims to develop a formulation for surface disinfection based on the combination of a natural product, cinnamaldehyde, and a widely used biocide, cetyltrimethylammonium bromide. The wiping method was based on the Wiperator test (ASTM E2967−15) and the efficacy evaluation of surface disinfection wipes test (EN 16615:2015). After formulation optimization, the wiping of a contaminated surface with 6.24 log_10_ colony-forming units (CFU) of *Escherichia coli* or 7.10 log_10_ CFU of *Staphylococcus aureus* led to a reduction of 4.35 log_10_ CFU and 4.27 log_10_ CFU when the wipe was impregnated with the formulation in comparison with 2.45 log_10_ CFU and 1.50 log_10_ CFU as a result of mechanical action only for *E. coli* and *S. aureus*, respectively. Furthermore, the formulation prevented the transfer of bacteria to clean surfaces. The work presented highlights the potential of a combinatorial approach of a classic biocide with a phytochemical for the development of disinfectant formulations, with the advantage of reducing the concentration of synthetic biocides, which reduces the potentially negative environmental and public health impacts from their routine use.

## 1. Introduction

Surface disinfection, when effective, is a part of a multibarrier strategy in fighting microbial contamination, such as preventing food spoilage and patient infection in healthcare settings. In fact, recently, the risk of foodborne transmission has increased due to consumer habits that include the consumption of raw vegetables and undercooking to retain the natural taste and to preserve heat-labile nutrients [1,2]. Pathogens such as *Klebsiella pneumoniae* have been detected on surfaces surrounding infected patients in healthcare settings with poor surface disinfection policies [3]. Despite the importance of surface disinfection, the choice of biocidal products (disinfectant) and disinfection frequency does not always depend on consequences for patients and staff if a pathogen remain uncontrolled on surfaces [4,5,6,7]. The use of disinfectants for general purposes is subject to controversies, despite the importance of surface disinfection being recognized [8,9,10,11,12,13]. One of the main concerns associated with the use of disinfectants is the development of biocide tolerance and/or cross-resistance to other biocides or even chemotherapeutic antibiotics as a consequence of the selective pressure exerted towards bacteria, especially when a biocidal product is misused [14,15]. Indeed, decreased bacterial susceptibility following exposure to suboptimal biocide concentrations, the use of an inappropriate biocide, or not following manufacturers’ guidelines, resulting in inappropriate product usage, has been documented [16,17,18].

There is scope for the development of new biocidal formulations with potent activity and decreased toxicity. The use of appropriate excipients that can work synergistically with the main biocide in order to increase the overall efficacy of a formulation provides an interesting avenue, notably to combat the rise of antimicrobial resistance [19,20]. The use of phytochemicals in particular offers a potential solution as an excipient [21]. This study focuses on cetyltrimethylammonium bromide (CTAB) and cinnamaldehyde. Quaternary ammonium compounds (QACs), such as CTAB, are among the most widely used biocides. CTAB is a cationic surfactant whose antibacterial activity is concentration-dependent. The positively charged form of CTAB interacts with the negative-charged bacterial membrane by nonselective electrostatic interactions. At low concentrations, it interferes with bacterial growth, inducing the cellular leakage of potassium and hydrogen ions, as well as loss of the ability for osmoregulation [22,23,24]. At higher concentrations, bacteria are killed due to the solubilization of the cellular membrane that ultimately leads to a fast leakage of cell components [22,23]. In addition, CTAB can generate superoxide and hydrogen peroxide and inhibit the regulatory gene *sosS* function and decrease manganese superoxide dismutase activity, leading to cell death [25].

Cinnamaldehyde occurs naturally in plants of the genus *Cinnamon* and is already known for its antimicrobial properties [26,27,28]. Cinnamaldehyde is already approved by the Food and Drug Administration of the United States and has the Generally Recognized as Safe status by the Flavor and Extracts Manufactures’ Association of the USA [29]. It can be used in air care products, perfumes and fragrances, polishes and waxes, washing and cleaning products, cosmetics and personal care products, and pharmaceuticals and biocides in the European Union [30].

The main goal of this work is to explore the possibility of a CTAB-cinnamaldehyde combination for the development of a formulation to be used with wipes for surface disinfection. To achieve this aim, the study was initiated using *Staphylococcus aureus* as a reference microorganism and *Escherichia coli* to corroborate the results achieved. The suspension test was used for formulation adjustments, and the evaluation of the efficacy of the final formulation was assessed through surface wiping.

## 2. Results

### 2.1. Formulation Optimization—Without Soil Load

**Figure 1:**
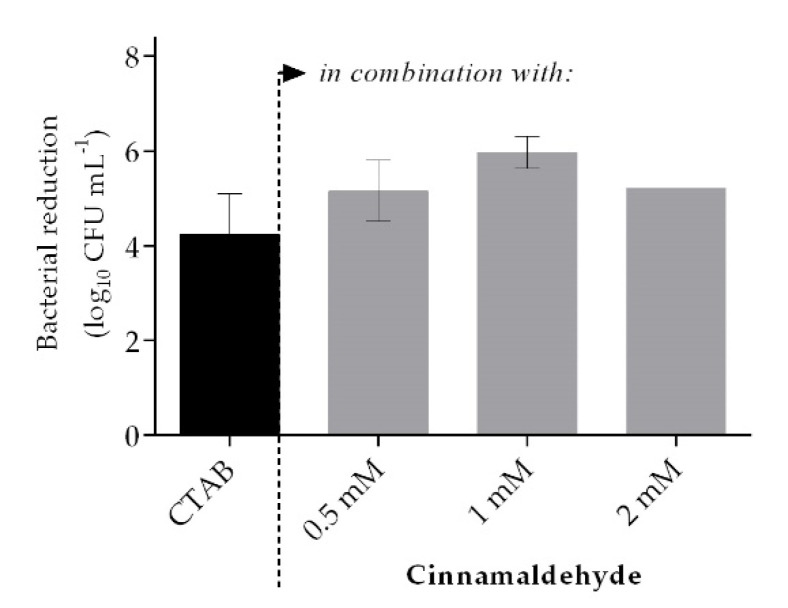
*Staphylococcus aureus* colony-forming unit (CFU) reduction after exposing a bacterial suspension for 5 min to 0.04-mM cetyltrimethylammonium bromide (CTAB) (black) alone or in combination (grey) with different concentrations of cinnamaldehyde. Values are mean ± SD. No statistical significance was observed between CTAB alone and any of the combinations (*p* > 0.05).

The efficacy of formulations combining QAC and a phytochemical were tested using the standardized efficacy test EN 1276:2009 [31]. A CTAB concentration of 0.04 mM, which allows a reduction in *S. aureus* of circa 5 log_10_ colony-forming units (CFU) mL^−1^, was selected to assess the impact of the phytochemical on the efficacy of the combination. CTAB was combined with different concentrations (0.5 mM, 1 mM, and 2 mM) of cinnamaldehyde. The CFU reduction obtained by the different concentrations of cinnamaldehyde alone was almost negligible (0.5 mM: 0.08 ± 0.04 log_10_ CFU mL^−1^, 1 mM: 0.07 ± 0.08 log_10_ CFU mL^−1^, and 2 mM: 0.05 ± 0.00 log_10_ CFU mL^−1^). After five-min contact, the combination CTAB-cinnamaldehyde showed a higher efficacy in comparison to CTAB alone. The best efficacy was achieved with 0.04-mM CTAB combined with 1-mM cinnamaldehyde (*p* > 0.05), resulting in a bacterial reduction of 5.97 ± 0.33 log_10_ CFU mL^−1^ (Figure 1).

**Figure 2:**
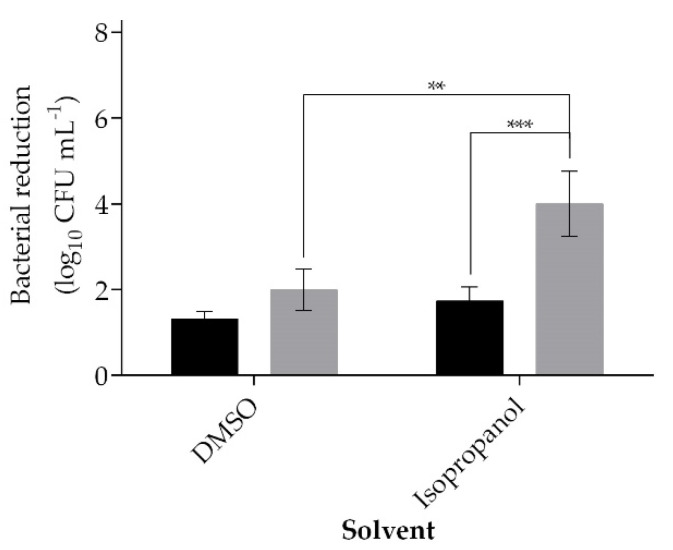
*S. aureus* reduction after exposing a bacterial suspension for 5 min to 0.02-mM CTAB (black) alone or in combination with 1 mM of cinnamaldehyde (grey) when two different solvents were used on the formulation (dimethyl sulfoxide (DMSO) or isopropanol). Values are mean ± SD. The statistical significance is represented (** *p* < 0.01 and *** *p* < 0.001).

The concentration of CTAB was reduced in half to better measure the impact of cinnamaldehyde when in combination, since a reduction of 4.25 ± 0.85 log_10_ CFU mL^−1^ was reached when CTAB was used alone (Figure 1). Two solvents, dimethyl sulfoxide (DMSO) and isopropanol were used to solubilize the phytochemical (maximum concentration of 5% *v v^−1^*). The combination of CTAB (0.02 mM)-cinnamaldehyde had a higher efficiency in comparison to CTAB alone, regardless the solvent used (Figure 2). The combination caused a reduction of 1.99 ± 0.48 and 4.00 ± 0.76 log_10_ CFU mL^−1^ whether cinnamaldehyde was dissolved in DMSO or isopropanol (*p* < 0.001), respectively. CTAB alone promoted a reduction of 1.31 ± 0.18 and 1.73 ± 0.33 log_10_ CFU mL^−1^ when in the presence of 5% *v v^−1^* of DMSO or isopropanol, respectively (Figure 2). Exposure of a *S. aureus* bacterial suspension for five min to 1 mM of cinnamaldehyde alone was reduced 0.05 ± 0.02 log_10_ CFU mL^−1^ if dissolved in DMSO or 0.04 ± 0.05 log_10_ CFU mL^−1^ when isopropanol was used. The CFU reduction obtained with the combination just by changing the solvent from DMSO to isopropanol was significantly higher (*p* < 0.01). Therefore, the subsequent studies were carried out using isopropanol as the cinnamaldehyde solvent.

**Figure 3:**
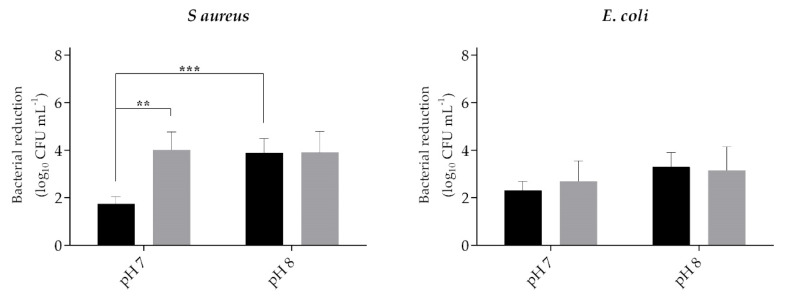
*S. aureus* and *Escherichia coli* CFU reduction after exposing a bacterial suspension for 5 min to 0.02-mM CTAB alone (black columns) or in combination with 1 mM of cinnamaldehyde (grey columns) when two different pH, pH 7 or pH 8, were used in the formulation. Values are mean ± SD. The statistical significance is presented (** *p* < 0.01 and *** *p* < 0.001).

The combination CTAB-cinnamaldehyde was also tested at pH 7 and 8 against *S. aureus* and *E. coli* (Figure 3). Exposing bacterial suspensions to 1 mM of cinnamaldehyde alone led to a reduction of 0.04 ± 0.05 log_10_ CFU mL^−1^ in *S. aureus* and 0.05 ± 0.06 log_10_ CFU mL^−1^ in *E. coli* at pH 7. At pH 8, a reduction of 0.02 ± 0.10 log_10_ CFU mL^−1^ in *S. aureus* and 0.06 ± 0.07 log_10_ CFU mL^−1^ in *E. coli* was observed. When the combination of CTAB and the phytochemical was assessed against *S. aureus* (Figure 3), the pH impacted the efficiency of CTAB alone. In this case, a reduction of 1.73 ± 0.33 log_10_ CFU mL^−1^ was achieved at pH 7, while a reduction of 3.89 ± 0.60 log_10_ CFU mL^−1^ (*p* < 0.001) was obtained for pH 8. However, when CTAB was combined with cinnamaldehyde, the *S. aureus* reduction at pH 7 was 4.00 ± 0.76 log_10_ CFU mL^−1^, while, at pH 8, was 3.90 ± 0.89 log_10_ CFU mL^−1^. Considering *E. coli* (Figure 3), it was possible to observe that the effect of CTAB alone was higher at pH 8 (*p* > 0.05). In addition, only at pH 7, it was observed a positive effect of the presence of 1 mM of cinnamaldehyde on *E. coli* reduction, from a 2.29 ± 0.40 log_10_ CFU mL^−1^ reduction of CTAB alone to a 2.69 ± 0.87 log_10_ CFU mL^−1^ reduction when combined with cinnamaldehyde (*p* > 0.05). A pH of 7 was selected for the formulation.

### 2.2. Formulation Optimization—Improving Activity against Gram-Negative Bacteria

To improve the efficacy of the formulation against the Gram-negative bacterium, ethylenediaminetetraacetic acid (EDTA) was added to the formulation. EDTA is an ion chelator that interacts with Ca^2+^-destabilizing lipopolysaccharides in the Gram-negative outer membrane, which results in increased permeability [32,33]. Accordingly, the screening of different concentrations of cinnamaldehyde, EDTA, and CTAB were performed (data not shown). A combination of 1-mM cinnamaldehyde, 25-mM EDTA, and 0.5-mM CTAB was chosen for further testing and optimization (Table 1).

### 2.3. Formulation Optimization—With Soil Load

**Figure 4:**
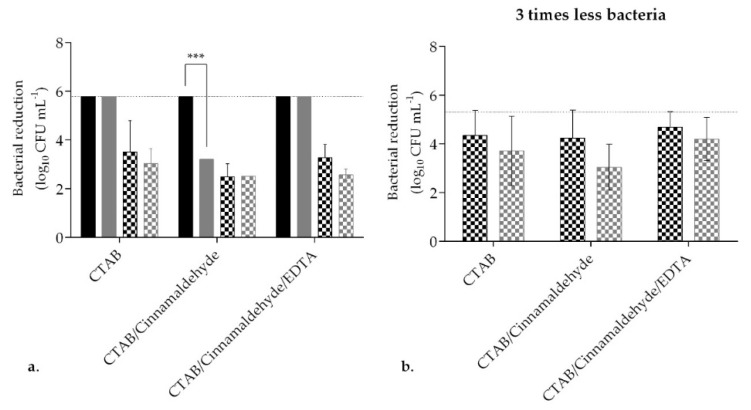
Bacterial reduction following exposure to formulations in the presence of soil load. (**a**). *S. aureus* (filled columns) and *E. coli* (pattern columns) CFU reductions after exposing a bacterial suspension for 5 min to CTAB alone, CTAB in combination with cinnamaldehyde, or CTAB in combination with cinnamaldehyde and ethylenediaminetetraacetic acid (EDTA). The test was performed under clean (black columns) or dirty (grey columns) conditions. (**b**). *E. coli* CFU reduction after exposing a 3-times less-concentrated bacterial suspension for 5 min to CTAB alone, CTAB in combination with cinnamaldehyde, or CTAB in combination with cinnamaldehyde and EDTA. The test was performed under clean (0.3 g L^−1^; grey columns) or dirty (3 g L^−1^; black columns) conditions. The horizontal dashed line in the figure represents a total reduction of bacteria considering the method limit of detection ((a) represented only for *S. aureus* 5.78 log_10_ CFU mL^−1^ and (b) 5.30 log_10_ CFU mL^−1^ for *E. coli*). Values are mean ± SD. The statistical significance is presented (*** *p* < 0.001).

A biocidal product needs to be active with and without the presence of soil load. To understand the performance of the formulation, both under clean (0.3 g L^−1^) and dirty conditions (3 g L^−1^), *S. aureus* and *E. coli* were exposed to CTAB, CTAB-cinnamaldehyde, and CTAB-cinnamaldehyde-EDTA (Figure 4). The efficacy of the formulation (CTAB-cinnamaldehyde-EDTA) against *S. aureus* was confirmed, as a total CFU reduction for clean and dirty conditions was observed, 5.78 log_10_ CFU mL^−1^ (Figure 4). The combination of CTAB-cinnamaldehyde was used under clean and dirty conditions, and a higher concentration of interfering substance decreased its efficacy against *S. aureus*, from 5.78 ± 0.00 to 3.20 ± 0.00 log_10_ CFU mL^−1^ (*p* < 0.001). When in contact with *E. coli,* the best results were obtained under clean conditions. The highest efficacy achieved for *E. coli* was observed for CTAB alone and CTAB-cinnamaldehyde-EDTA, with reductions of 3.50 ± 1.29 log_10_ CFU mL^−1^ and 3.27 ± 0.54 log_10_ CFU mL^−1^, respectively. When the bacterial suspension concentration was reduced to one-third (Figure 4), CTAB-cinnamaldehyde-EDTA showed a higher reduction of 4.69 ± 0.64 log_10_ CFU mL^−1^ and 4.20 ± 0.89 log_10_ CFU mL^−1^ under clean and dirty conditions, respectively (*p* > 0.05). At this point, it was decided to use the following formulation for the subsequent studies: 1-mM cinnamaldehyde, 25-mM EDTA, 0.5 mM CTAB in phosphate buffer (PB) at pH 7, and with 5% *v v^−1^* isopropanol.

### 2.4. Surface Wiping—Mechanical and Formulation Efficacy

**Figure 5:**
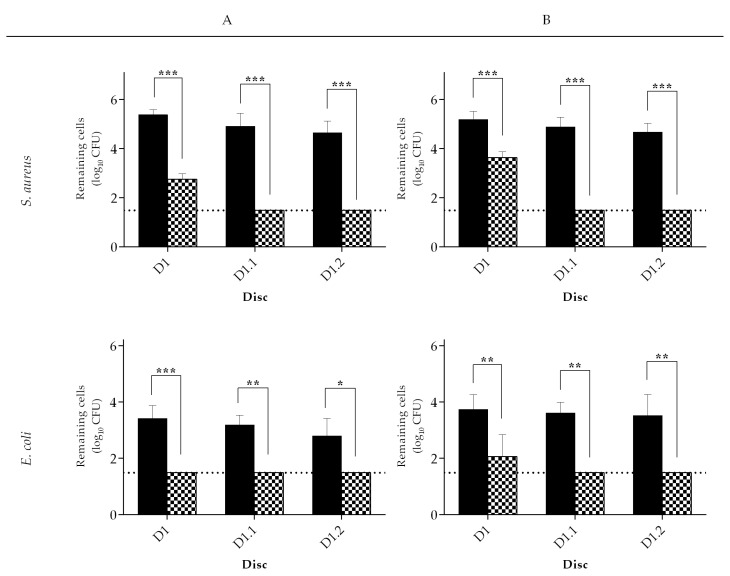
Remaining CFU on the contaminated surface (D1) and on two clean surfaces (D1.1 and D1.2) after being wiped with wipe **A** or **B**. *S. aureus* and *E. coli* were used as surfaces contaminants. Mechanical (filled columns) and biocidal (columns with pattern) actions were evaluated. Values are mean ± SD. Horizontal dashed line represents the limit of detection of the method (1.49 log_10_ CFU). The statistical significance is represented (* *p* < 0.05, ** *p* < 0.01, and *** *p* < 0.001).

As this formulation was to be used in combination with wipes, it is important to measure the efficacy of the product (formulation-wipe) rather than just the formulation alone [34]. To deliver the formulation, two different types of wipe materials were chosen (wipe A and B; Supplementary Materials A.3.). With the surface-wiping method, it was possible to study the ability to remove and kill bacteria from a contaminated surface (D1) and bacterial transfer to clean surfaces (D1.1 and D1.2). On their own, both wipe materials had the same effect (*p* > 0.05) on *S. aureus* removal from surfaces (Figure 5). In the contaminated surface D1, after wiping with wipe A, 5.38 ± 0.20 log_10_ CFU remained in the disc and, with wipe B, 5.19 ± 0.33 log_10_ CFU. While, in D1.1, 4.91 ± 0.52 log_10_ CFU and 4.88 ± 0.40 log_10_ CFU remained on the surface, in D1.2, 4.65 ± 0.47 log_10_ CFU and 4.68 ± 0.36 log_10_ CFU remained after using wipe A or B, respectively. When the wipes were impregnated with the formulation, a significant reduction (*p* < 0.001) of *S. aureus* on the surfaces was observed; only 2.76 ± 0.22 log_10_ CFU (wipe A) and 3.64 ± 0.24 log_10_ CFU (wipe B) remained on the surface, while the number of CFU on D1.1 and D1.2 were below the detection limit regardless of the wipe used. *E. coli* removal from surfaces (without formulation) was similar (*p* > 0.05) for both wipes (Figure 5). In fact, when wipe A was used, 3.42 ± 0.46 log_10_ CFU remained on the surface and, with wipe B, 3.73 ± 0.53 log_10_ CFU. As with *S. aureus*, the wipes on their own were not very effective (<log reduction) in reducing the number of *E. coli* on surfaces. In fact, when wipe A was used, D1.1 showed a contamination of 3.18 ± 0.35 log_10_ CFU and D1.2 of 2.80 ± 0.62 log_10_ CFU and, with wipe B, D1.1 a contamination of 3.61 ± 0.38 log_10_ CFU and D1.2 of 3.52 ± 0.74 log_10_ CFU. When the wipes were impregnated with the formulation, a significant decrease in *E. coli* concentration on the surfaces was observed (Figure 5). In fact, for wipe A, the CFU were below the detection limit, 1.49 log_10_ CFU, on D1 (*p* < 0.001), D1.1 (*p* < 0.01), and D1.2 (*p* < 0.05) when compared to the wipe without formulation. When wipe B was impregnated with the formulation, a 2.05 ± 0.79 log_10_ CFU remained on D1 (*p* < 0.01), and the CFU were below the limit of detection on D1.1 (*p* < 0.01) and on D1.2 (*p* < 0.01) in comparison to the wipe without the formulation.

**Figure 6:**
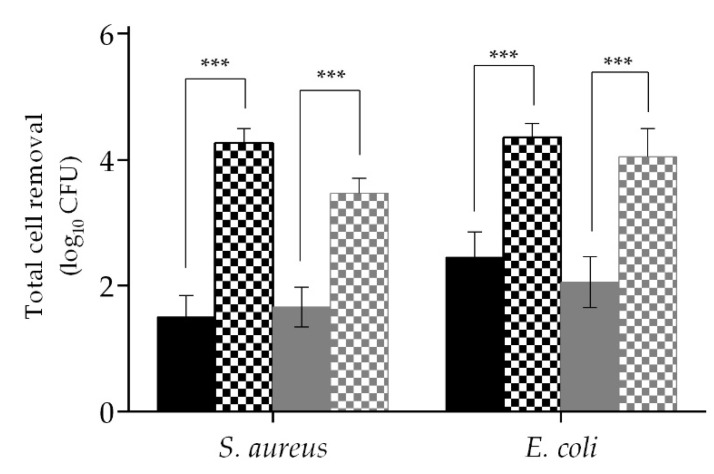
*S. aureus* and *E. coli* reduction of all the bacteria that were not recovered from the discs (total from surfaces). The mechanical action (filled columns) and the biocidal effect (columns with patterns) were tested for two types of wipes, A (black) and B (grey). Values are mean ± SD. The statistical significance is represented (*** *p* < 0.001).

A summary of the effectiveness of wipe A and B on the overall cell removal (bacteria that were removed and killed during the process of wiping three surfaces) is shown in Figure 6. In total, wipe A mechanical action removed from the surface 1.50 ± 0.35 log_10_ CFU of *S. aureus* and 2.45 ± 0.41 log_10_ CFU of *E. coli,* while wipe B removed 1.66 ± 0.31 log_10_ CFU of *S. aureus* and 2.06 ± 0.41 log_10_ CFU of *E. coli*. The efficacy of the wipe was improved when impregnated with the formulation. In fact, the impregnated wipe A was able to remove 4.27 ± 0.22 log_10_ CFU of *S. aureus* (*p* < 0.001) and 4.35 ± 0.22 log_10_ CFU of *E. coli* (*p* < 0.001), while wipe B removed 3.47 ± 0.23 log_10_ CFU of *S. aureus* (*p* < 0.001) and 4.04 ± 0.46 log_10_ CFU of *E. coli* (*p* < 0.001).

### 2.5. Formulation Chemical Stability

**Figure 7:**
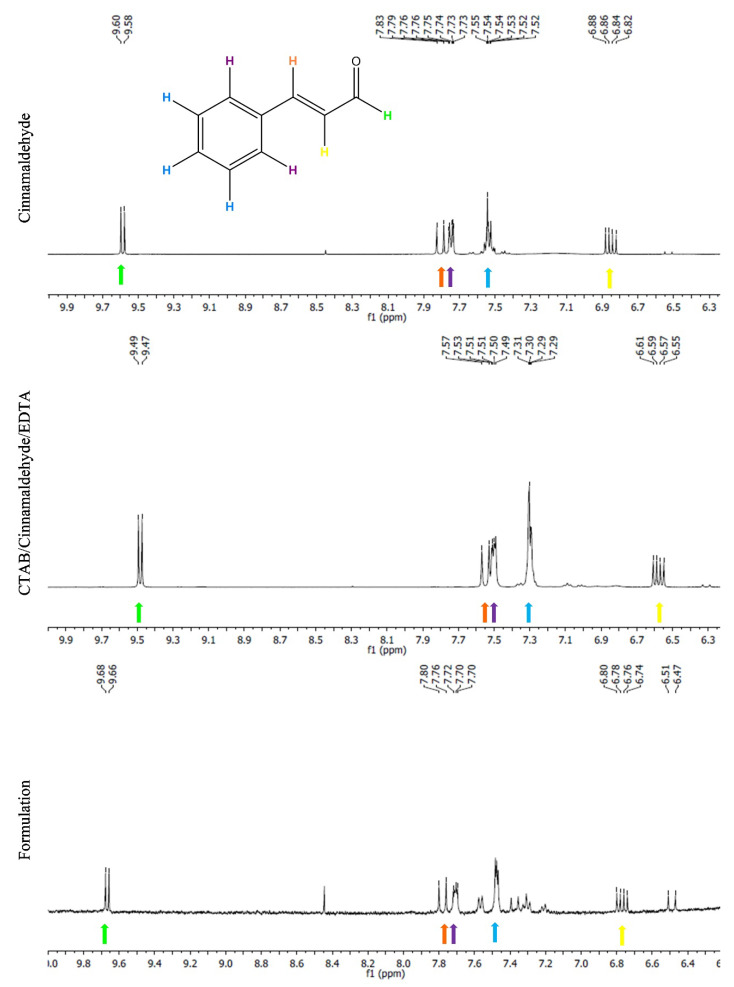
^1^H NMR spectra of cinnamaldehyde (top), a mixture of CTAB-cinnamaldehyde-EDTA in water (middle), and the formulation (bottom). All the solutions were prepared in deuterated water.

A preliminary study was performed to check for the potential chemical interaction of the phytochemical with the biocide or other components of the formulation. From the analysis of the ^1^H NMR spectra (Figure 7), it was possible to conclude that cinnamaldehyde maintains its chemical integrity as part of a mixture with CTAB-EDTA, whether in water or as a formulation. The same type of signals was observed in the comparative analysis with the spectrum of cinnamaldehyde.

## 3. Discussion

Inefficient cleaning and disinfection will contribute to surface contamination and microbial spreading, which, overall, may impact on the infection rate [8,35]. There are many protocols to impart cleaning or disinfectant formulations on surfaces, but the majority use some form of wiping [34,36]. To ensure microbicidal efficacy, a biocidal product needs to be tested in accordance with official standards, such as the EN 1276:2009 “Chemical disinfectants and antiseptics” in Europe [31]. According to this particular standard test, a reduction of 5 log_10_ CFU mL^−1^ must be achieved. In this work, we explored a formulation combining both CTAB and cinnamaldehyde, which was found to be synergistic in our previous work [21,37,38]. Initially, the combination of CTAB (0.04 mM) with 1 mM of cinnamaldehyde was found to be an efficient bactericidal, killing more than 5 log_10_ CFU mL^−1^ within a five-min contact time (Figure 1). CTAB is a cationic surfactant whose mode of action is related to the denaturation of proteins, inducing changes in the properties of the cell membrane and facilitating the entrance of other antimicrobials into the bacterial cell [24]. This cationic surfactant is also able to concentrate, solubilize, and compartmentalize ions and molecules, a process that can enhance its antimicrobial action and, also, of other antimicrobials [39]. In this work, it was hypothesized that the combined effect of CTAB with cinnamaldehyde may be advantageous, as this phytochemical has a low MW (132.162 g mol^−1^) and lipophilicity, which are parameters that can facilitate cinnamaldehyde permeation across the bacterial membrane [27,40]. Cinnamaldehyde is described to be able to react with membranes, proteins, nucleic acids, lipids, and carbohydrates of *E. coli* [41]. It was also hypothesized that low concentrations of cinnamaldehyde can act on the cell membrane components, and, at a higher dosage, it can diffuse into the bacteria, modifying the cytoplasm enzymes in the transcriptome and, consequently, promoting cell death [40,42,43,44]. At a reduced concentration of 0.2-mM CTAB, the formulation did not perform as well against Gram-negative compared to Gram-positive. The use of isopropanol as a solvent and a chelator (EDTA) improved the efficacy of the formulation (Figure 2 and Figure 4). The difference in antimicrobial susceptibility between Gram-negative and Gram-positive bacteria has been well-reported [33,45,46]. The chelator EDTA was used to increase the efficacy of the formulation in Gram-negative bacteria, as it disrupts the lipopolysaccharide structure in the outer membrane of Gram-negative bacteria [16,46]. Some authors hypothesized that the presence of EDTA can cause potentiation as a result of a loss of barrier function of the outer membrane, such as efflux pumps inhibition, as well as an enhanced uptake mechanism or removal of inactivating factors that are found in the membrane or in the periplasmic space [32,46,47]. The use of EDTA has been reported as a potentiator of the activity of antimicrobials, antibiotics, preservatives, and cationic surfactants such as QACs [32,33,47,48]. In fact, when combined with a QAC, EDTA even demonstrated synergy against *Pseudomonas aeruginosa*, and an inhibition model of the EDTA mode of action was suggested [46]. One happens below a threshold concentration and, according to the literature, is a general inhibitory process, such as removing metal ions from the growth medium, while the second, that happens at a higher concentration, corresponds to the destabilization of the outer membrane that, consequently, leads to cell lysis.

The presence of an organic load can impact negatively the efficacy of a formulation [16,17], and indeed, the activity of the formulation developed (1-mM cinnamaldehyde, 25-mM EDTA, and 0.5-mM CTAB in PB pH 7 with 5% *v v^−1^* isopropanol) was reduced (Figure 4). Wipes, cloths, etc. are usually used in combination with a formulation [34]. One advantage of prewetted wipes is the additional mechanical effect contributing to the overall reduction of microorganisms from the surface. The importance of testing a final product is reflected with the use of standard efficacy tests such as the Wiperator test (ASTM E2967−15) or the efficacy evaluation of surface disinfection wipes test (EN 16615-15) [49,50]. The combination of the formulation with wipe material improved the overall efficacy of the product while preventing the transfer of bacteria to other surfaces (Figure 5). Measuring the ability to prevent bacterial transfer together with a reduction in bacterial viability provides a better understanding of the effectiveness of biocidal products to render a safe surface [51,52].

In this study, we showed that a promising synergistic effect between CTAB and cinnamaldehyde [32,33,34] can be translated into a formulation that, when combined with a wipe material, can deliver bactericidal efficacy on surfaces. This, in turn, opens up the prospect of using phytochemicals as excipient to improve the effectiveness of a biocidal product.

## 4. Materials and Methods

### 4.1. Chemicals and Test Solutions

CTAB (CAS: 57-09-0), was purchased from Acros Organics (Portugal), while cinnamaldehyde (CAS: 14371-10-9) and bovine serum albumin (BSA) were purchased from Sigma Aldrich (Portugal). EDTA was acquired from Panreac. Lecithin, polysorbate 80, thiosulphate, saponin, isopropanol, and DMSO were obtained from VWR Chemicals. L-histidine was purchased from Merck. All reagents were of analytical grade. Solutions of CTAB, BSA, and EDTA were prepared in sterile deionized water and cinnamaldehyde in DMSO or isopropanol. The biocide and phytochemical neutralization step was performed using the universal neutralizer (lecithin 3 g L^−1^, polysorbate 80 30 g L^−1^, thiosulphate 5 g L^−1^, L-histidine 1 g L^−1^, and saponin 30 g L^−1^ in 1% phosphate buffer 0.25 M, pH 7.2) for 10 min, which was shown to be efficient in inactivating the biocidal activity and to be nontoxic to the test bacteria (data not shown).

### 4.2. Microorganisms, Culture Conditions, and Test Solutions

*Staphylococcus aureus* NCTC 10788 and *Escherichia coli* NCTC 10418 were used in this study. Test suspensions were obtained from overnight cultures in 100-mL flasks with 25 mL of Mueller-Hinton broth prepared in phosphate buffer (0.02 M pH 7 and PB pH 7) incubated at 37 °C and under 150 rpm agitation on an orbital shaker (25 mm of orbital radius, Agitorb 200ICP), as described previously [21,38].

### 4.3. Bactericidal Suspension Test

The suspension test used was adapted from the EN 1276:2009 [31]. Briefly, the overnight culture was washed once with PB, pH 7, and the bacterial suspension was adjusted to an OD_600_ nm of 0.33 with PB, pH 7 (1.5–5 CFU mL^−1^). A volume of 900 µL of cell suspension was added to an Eppendorf containing cinnamaldehyde (5% *v v^−1^* of solvent and 0.5 mM, 1 mM, and 2 mM of cinnamaldehyde) and CTAB (from 0.005 to 1 mM) for a total of 1 mL of test solution and vortexed for 5 s. Before adding the bacterial cells, the test solution was incubated statically for 2 min at 25 ± 3 °C. Incubation at 25 ± 3 °C was allowed to occur for 5 min. A volume of 100 μL of the test solution was placed in 900 μL of neutralizer for 10 min, and CFU determination was performed. CFU were determined after 24 h at 30 °C incubation and presented as log_10_ CFU cm^−2^. Three independent experiments were performed for each condition tested.

To test the impact of the organic load, 500 µL of cell suspension (OD_600_ nm at 0.6) were added to an Eppendorf containing a phytochemical/derivative (5% *v v^−1^* of solvent and 1 mM or 2 mM of cinnamaldehyde final concentration), CTAB (0.02 to 1 mM), EDTA (10 mM or 25 mM), BSA (0.3 g L^−1^ or 3 g L^−1^ for clean or dirty conditions), and 250 µL of PB, pH 7, for a total of 1 mL of test solution. The results are presented as log_10_ CFU mL^−1^.

### 4.4. Surface Wiping Assay

A surface wiping assay protocol was developed for the accomplishment of this data (for more information, see Supplementary Materials A.1., A.2., and A.3.).

#### 4.4.1. Preparation of the Contaminated Surface D1

An overnight culture was washed twice with PB pH 7, and the cell suspension was adjusted to 1.5–5 × 10^9^ CFU mL^−1^ (Supplementary Materials A.1.) [49,50]. Prior to using the cell suspension, 0.5 mL of ten-times concentrated BSA (final concentration 0.3 g L^−1^) was added to 4.5 mL of bacterial suspension and vortex for 30 s. A volume of 10 µL of this suspension was transferred to the center of a clean and sterile stainless-steel disc (2-cm diameter) and allowed to completely dry at 37 ± 3 °C for 30 min (D1).

#### 4.4.2. Preparation of the Wipe Carrier 

The control solution (0.1% polysorbate 80 in water) and the formulation (1-mM cinnamaldehyde dissolved in 5% *v v^−1^* isopropanol, 0.5-mM CTAB, and 25-mM EDTA in 20-mM PB, pH 7) were freshly prepared for each experiment. The wipe (4 × 4 cm^2^) was presoaked in 20 mL of solution, ensuring the wipe was completely covered for 2 min at room temperature (25 ± 3 °C). With a clean pair of gloves, the wipe was wrung to drain the excess of liquid and weighted after removal of the excess liquid: wipe A—0.233 ± 0.030 g and 0.214 ± 0.031 g and wipe B—0.370 ± 0.019 g and 0.355 ± 0.017 g for the control and formulation, respectively. The wipe was then wrapped onto the carrier and fixed with a rubber band (Supplementary Materials A.2.).

#### 4.4.3. Wiping Test

**Figure 8:**
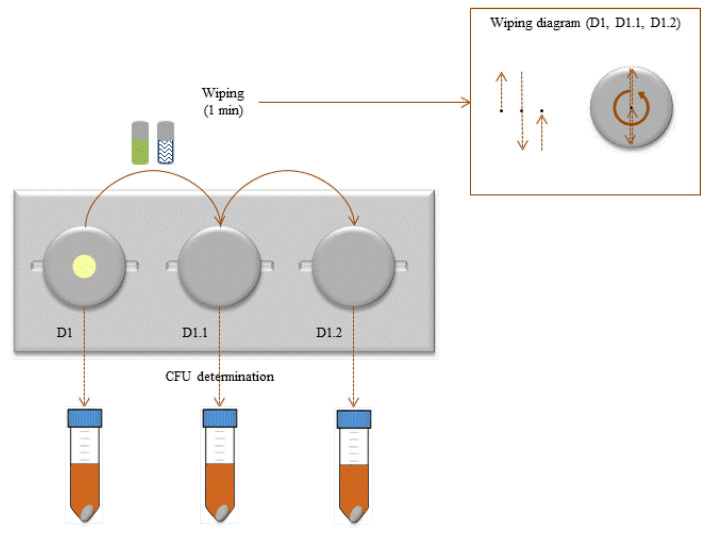
Schematic representation of the wiping assay steps.

The wiping test was performed as presented in Figure 8. Briefly, D1 was placed on the holder with two clean and sterile stainless-steel discs next to it. The wipe carrier was placed on the top of D1, and with the help of forceps, the wipe carrier was moved (without putting any pressure on the wipe carrier) towards the disc for 1 min vertically and horizontally, as described in the diagram showed in Figure 8. The wipe carrier was then moved to D1.1 and D1.2, repeating the surface wiping movement for 1 min for each disc (Figure 8). After wiping of all three discs, each disc was placed in 5 mL of universal neutralizer with 2 g of glass beads and vortexed for 5 s. Neutralization was allowed to occur for 10 min. Discs were then vortexed for 30 s, and CFU determination was performed as described before. The results are presented as a log_10_ CFU mL^−1^ reduction or log_10_ CFU.

### 4.5. Evaluation of Phytochemical/Biocide Chemical Interaction Study by Nuclear Magnetic Resonance Spectroscopy

^1^H NMR (nuclear magnetic resonance) data were acquired on a Bruker Avance III 400 NMR spectrometer operating at 400.15 MHz. The relaxation delay was 90° pulse, a spectral width of 8012 Hz, and 65 K data points. ^1^H NMR spectra of the samples were recorded at room temperature (25 ± 3 °C) in 5-mm outer diameter tubes. The samples were prepared in deuterated water. TMSP-*d4* (3-(trimethylsilyl) propionic-2,2,3,3-*d4* acid sodium salt) was used as the internal reference.

### 4.6. Statistical Analysis

The statistical program GraphPad Prism version 6 was used to analyze the data. One-way analysis of variance (one-way ANOVA) followed by the post hoc Dunnett’s multiple comparison test. Confidence levels of ≥95% (*p* < 0.05), ≥99% (*p* < 0.01), and ≥99.9% (*p* < 0.001) were used to consider statistical significance. The results were presented as the average and standard deviation (SD) of three independent experiments for each sample.

## 5. Conclusions

In this study, we showed that a promising synergistic effect between CTAB and cinnamaldehyde can be translated in a formulation that, when combined with a wipe material, can deliver bactericidal efficacy on surfaces. This, in turn, opens up the prospect of using phytochemicals as excipients to improve the effectiveness of a biocidal product. 

## Figures and Tables

**Table 1:** Concentrations tested for cinnamaldehyde, EDTA, and CTAB in combination by using the suspension test against *Escherichia coli* NCTC 10418. EDTA: ethylenediaminetetraacetic acid and CTAB: cetyltrimethylammonium bromide.

Cinnamaldehyde (mM)	EDTA (mM)	CTAB (mM)
1	10	0.02
	25	0.02 < (CTAB) < 0.5
		0.5
		0.5 < (CTAB) < 1
		1

## Supplementary Materials

The following are available online at https://www.mdpi.com/1422-0067/21/21/7852/s1.

## Abbreviations

BSA	Bovine serum albumin
CFU	Colony-forming units
CTAB	Cetyltrimethylammonium bromide
DMSO	Dimethyl sulfoxide
EDTA	Ethylenediaminetetraacetic acid disodium salt
NMR	Nuclear magnetic resonance
PB	Phosphate buffer
QAC	Quaternary ammonium compound
