# Supplementary material for: Surface Wiping Test to Study Biocide -Cinnamaldehyde Combination to Improve Efficiency in Surface Disinfection
Source: Int J Mol Sci. 2020 Oct 23;21(21):7852. doi: 10.3390/ijms21217852 (PMC7660177; doi:10.3390/ijms21217852)
Supplement: Supplementary file 1 [file ijms-21-07852-s001.pdf]

International Journal of *Molecular Sciences*

MDPI

Supplementary Materials

Surface wiping test to study biocide - cinnamaldehyde combination to improve surface disinfection efficiency

Joana F. Malheiro [1,2,3], Catarina Oliveira [3], Fernando Cagide [3], Fernanda Borges [3], Manuel Simões [1*], Jean-Yves Maillard [2*]

1 LEPABE - Laboratory for Process Engineering, Environment, Biotechnology and Energy, Faculty of Engineering, University of Porto, Rua Dr. Roberto Frias, 4200-465 Porto, Portugal
2 Cardiff School of Pharmacy and Pharmaceutical Sciences, Cardiff University, Cardiff, Wales CF10 3NB, United Kingdom
3 CIQUP, Department of Chemistry and Biochemistry, Faculty of Sciences University of Porto, Rua do Campo Alegre, 4169-007 Porto, Portugal
* Correspondence: e-mail: mvs@fe.up.pt (MS), maillardj@cardiff.ac.uk (JYM); Tel.: +351225081654 (MS), +44 (0)29 2087 9088/+44 (0)29 2087 4149 (JYM).

A. Surface wiping assay

A.1. Bacterial Suspension – $OD_{600\ nm}$ optimization

According to the Standards used as reference to assess bactericidal activity when pre-wetted wipes are used [1,2], a bacterial suspension of 1.5×10^9 CFU mL^{-1} to 5.0×10^9 CFU mL^{-1} needs to be used to inoculate the discs and at least $10^{5.5}$ CFU disc^{-1} should be recovered after drying. Taking into consideration the results obtained (Table S1) a suspension OD_{600nm} of 4 and 2 were selected for *E. coli* and *S. aureus* and a drying time of 30 min at 37 ± 3 ºC.

Table S1: Bacterial suspension concentration at different optical density (OD_{600nm}) of *E. coli* and *S. aureus*. The drying time was also optimized when an initial OD_{600nm} of 4 and 2 were used for *E. coli* and *S. aureus*

	E. coli			*S. aureus*		
**OD_{600nm}**						
**1**	1.03×10^9	±	2.18×10^8CFU mL^{-1}	8.25×10^8	±	1.25×10^8 CFU mL^{-1}
**1.6**	3.42×10^9	±	3.82×10^8 CFU mL^{-1}	1.98×10^9	±	4.62×10^8 CFU mL^{-1}
**2**		-		1.43×10^9	±	3.51×10^8 CFU mL^{-1}
**3**	3.92×10^9	±	1.38×10^9 CFU mL^{-1}		-	
**4**	6.13×10^9	±	2.92×10^9 CFU mL^{-1}		-	
**Drying time**						
**30 min**	1.74×10^6	±	8.55×10^5 CFU	1.27×10^7	±	1.76×10^6 CFU
**45 min**	1.17×10^6	±	1.42×10^5 CFU	7.67×10^6	±	1.91×10^6 CFU
**1 h**	6.50×10^5	±	2.50×10^4 CFU	5.17×10^6		1.66×10^6 CFU

A.2. Holder and carrier development

The holder and carrier were designed based on EN 16615:2015 - Efficacy evaluation of surface disinfection Wipes, where a block of 2.3 – 2.5 Kg (18.6 × 12.1 × 8.6 cm) is used to mimic the pressure that is used when a surface is cleaned using a wipe [1]. In addition, this design was intended to be a smaller scale, where a larger number of samples could be assessed in a shorter period of time at low cost. Therefore, a disc system was developed taking as an example the Wiperator (E2967 − 15) sampling system [2].

The size and weight of the carrier was determined by using the formula of the area (5), the force (6), pressure (7), density (8) and volume (9):

$$\text{Area}_{\text{rectangle}} = \text{height} \times \text{width};\ \text{Area}_{\text{circle}} = \pi \times \text{radius}^2 \quad (5)$$

$$\text{Force} = \text{mass} \times \text{acceleration} \quad (6)$$

$$\text{Pressure} = \text{force} \div \text{area} \quad (7)$$

$$\text{density} = \text{mass} \div \text{volume} \quad (8)$$

$$\text{Volume} = \pi \times \text{radius}^2 \times \text{height} \quad (9)$$

Therefore, taking into consideration the values for the granite block, the pressure that the block exerts to the surface needs to be obtained in order to have a carrier that exerts exactly the same pressure to the disc ($P_{\text{block}} = P_{\text{carrier}}$). In addition, the radius of the carrier should be half the disc (0.5 cm):

$A_{\text{block}} = L \times w = 0.121 \times 0.086 = 0.0104\ m^2$

$F_{\text{block}} = m \times a = 2.3 \times 9.8 = 22.5$ N (2.3 Kg)
$= 2.5 \times 9.8 = 24.5$ N (2.5 Kg)

$P_{\text{block}} = F \div A = 2166$ Pa (2.3 Kg)
$= 2354$ Pa (2.5 Kg)

$A_{\text{carrier}} = \pi \times r^2 = \pi \times (0.005)^2 = 0.0000785\ m^2$

$F_{\text{carrier}} = P \times A = 0.1701$ N (2.3 Kg)
$= 0.1849$ N (2.5 Kg)

$m_{\text{carrier}} = F \div a = 0.0173$ Kg (2.3 Kg)
$= 0.0189$ Kg (2.5 Kg)

$d = m \div V \Leftrightarrow 8\ g\ cm^{-2}$ (stainless steel) $= m \div V \Leftrightarrow V_{\text{carrier}} = 2.17\ cm^3$ (2.3 K g)
$= 2.36\ cm^3$ (2.5 Kg)

$V_{\text{carrier}} = \pi \times r^2 \times h \Leftrightarrow$ **$h_{\text{carrier}} = 2.76$ cm (2.3 Kg)**

$= 3.00$ cm (2.5 Kg)

In Figure S1 it is possible to see the design of the holder and carrier considering all the information described above.

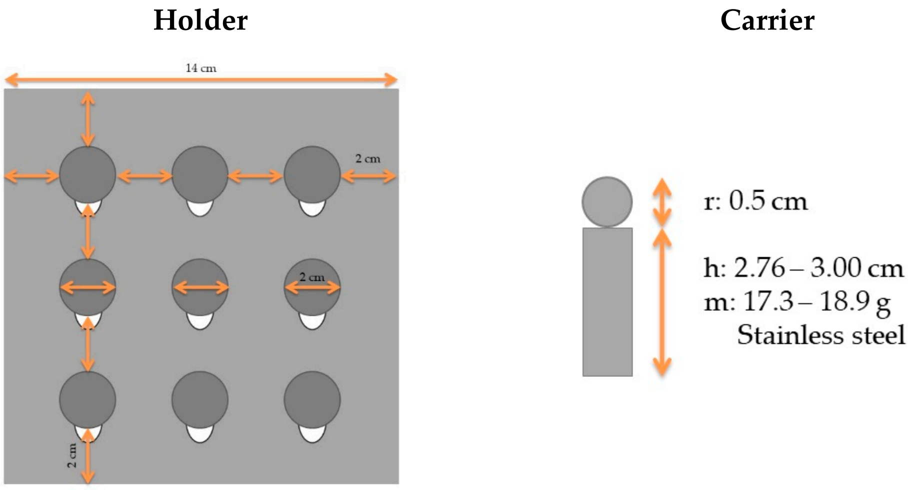

Figure S1: Holder (left) and carrier (right) design in 2D.

All the components for this assay are presented in Figure S2. The wipes were purchased from Bastos Viegas SA (Penafiel, Portugal). The holder for the discs, the discs and the carrier were done in stainless steel AISI 316.

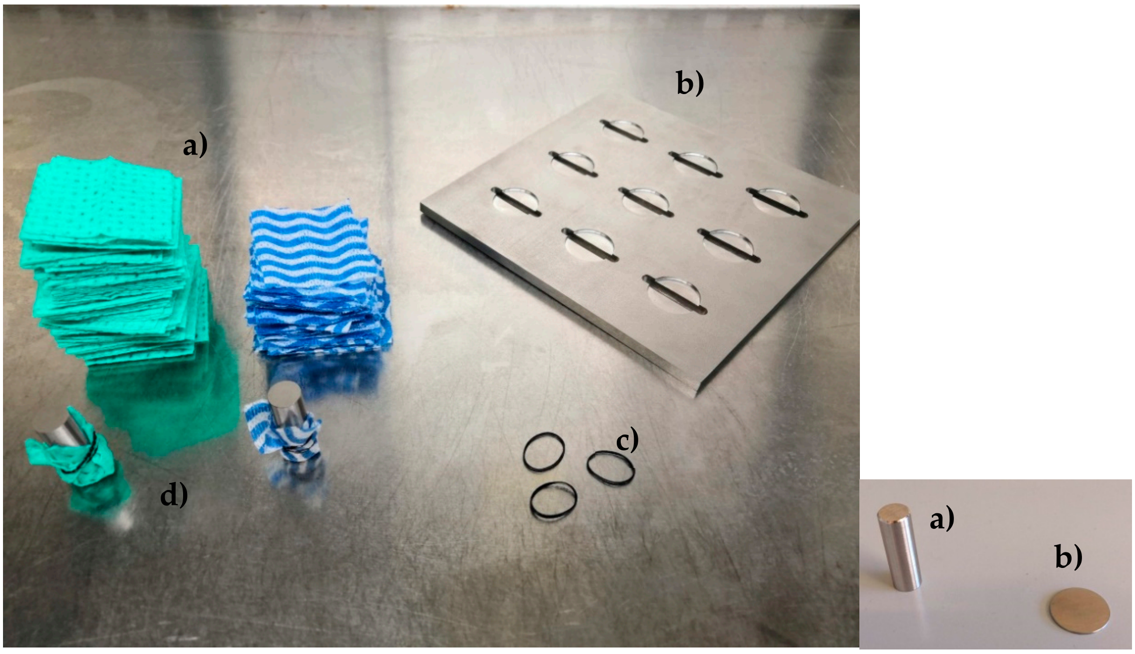

Figure S2: Components of the surface wiping efficiency test. On the left: a) wipes, b) holder for the discs, c) rubber and d) wipe carrier. On the right: a) carrier, b) stainless steel disc.

A.3. Wipe characterization

The wipes that were purchased from Bastos Viegas SA (Penafiel, Portugal) had different characteristics, such as composition and structure Figure S3. The wipes used in this work were 90% fibres (60% viscose/40% polyester) and 10% synthetic ligand named wipe A (white/blue) and wipe B (green) had 94% de fibres (70% viscose/30% polyester) and 6% synthetic ligand. Using optical coherence tomography (Thorlabs Ganymede Spectral Domain OCT system with central wavelength of 930 nm, Thorlabs GmbH, Dachau, Germany, using a LSM03 objective lens (5 × magnification)) it was possible to obtain some data on the thickness and pore diameter of both wipes that is presented on Table S2.

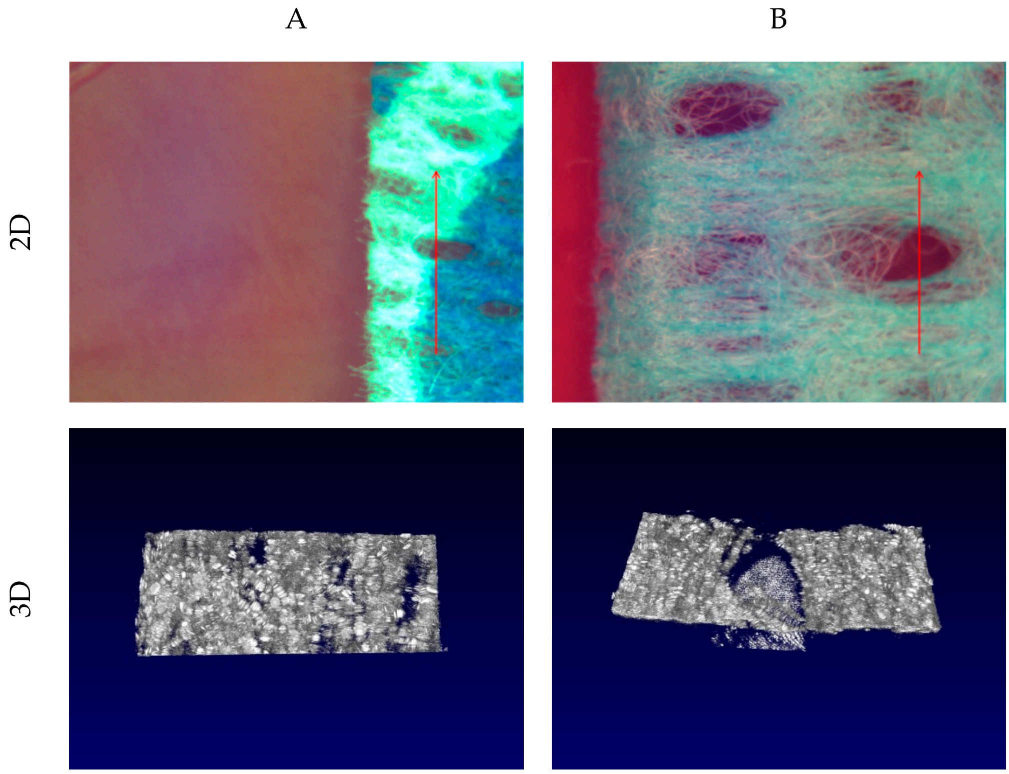

Figure S3: Optical coherence tomography (OCT) 2D and 3D and images of wipe A (right) and B (left) 5 times amplification.

Table S2: OCT measurements of wipe thickness and pore diameter based on the section illustrated in Figure S3

	**A**	**B**
Thickness (mm)	0.22 ± 0.04	0.28 ± 0.06
Pore diameter (mm)	0.42 ± 0.18	0.98 ± 0.06

References

1. CEN. EN 16615 Chemical disinfectants and antiseptics - Quantitative test method for the evaluation of bactericidal and yeasticidal activity on nonporous surfaces with mechanical action employing wipes in the medical area (4- field test) - Test method and requirements (phase 2, step 2). 2015.
2. ASTM. E2967 − 15 Standard test method for assessing the ability of pre-wetted towelettes to remove and transfer bacterial contamination on hard, non-porous environmental surfaces using the Wiperator. 2016.

© 2020 by the authors. Submitted for possible open access publication under the terms and conditions of the Creative Commons Attribution (CC BY) license (http://creativecommons.org/licenses/by/4.0/).
